# Learning to use music as a resource: the experiences of people with dementia and their family care partners participating in a home-based skill-sharing music intervention: a HOMESIDE sub-study

**DOI:** 10.3389/fmed.2023.1205784

**Published:** 2023-05-18

**Authors:** Kate McMahon, Katrina McFerran, Imogen N. Clark, Helen Odell-Miller, Karette Stensæth, Jeanette Tamplin, Felicity A. Baker

**Affiliations:** ^1^Faculty of Fine Arts and Music, The University of Melbourne, Melbourne, VIC, Australia; ^2^Cambridge Institute for Music Therapy Research, Anglia Ruskin University, Cambridge, United Kingdom; ^3^Centre for Research in Music and Health, Norwegian Academy of Music, Oslo, Norway

**Keywords:** music therapy, dementia, family caregiver, caregiving dyad, skill-sharing, family care partner, living in the community, indirect music therapy

## Abstract

An increasing number of people with dementia receive informal care from family members to help them remain living in the community. Music therapy is particularly beneficial for supporting the wellbeing of people living with dementia. However, little is known about how music therapy might support people with dementia and their family care partners as dyads. This study explored the experiences of six dyads participating in a 12-week home-based skill-sharing music intervention facilitated by a music therapist. We examined their experiences during the intervention period and in the 3–6 months following. This study was conducted within a larger randomised control trial, HOMESIDE. Data was collected through video-recorded music-based interviews, participant diaries, and a semi-structured interview. Data was analysed using an abductive and relational-centred research approach in consideration of the *Contextual Connection Model of Health Musicking for People Living with Dementia and Their Family Care Partners*. The study found fifteen themes that describe dyads’ supported experiences of sharing music in their homes. These were organised into three global themes: (1) experiences were shaped by complex influences; (2) a connected musical ecosystem; and (3) music was a resource for wellbeing. This study highlighted the important role of personalised facilitation and the therapeutic relationship as dyads learned to use music as a resource through a process of trial and error. The implications for skill-sharing, indirect music therapy and direct music therapy practice are discussed.

## 1. Introduction

Dementia is a global health priority with more than 55 million people currently living with a diagnosis and cases increasing by nearly 10 million per year (1). To support people with dementia to remain at home, family members and friends provide increasing amounts of informal care (1). While providing care can be fulfilling, it also presents numerous challenges (2) and is a risk factor for stress (3). Within caring relationships, the wellbeing of the person with dementia and their family care partners as dyads is interconnected (4, 5). In light of this, dyadic approaches to care are increasingly recommended to support dyads individually and collectively (4, 6). Therapeutic music interventions, including music therapy, are uniquely helpful for people living with dementia, supporting memory, mood, communication and social connection (7, 8). There is an emerging body of research exploring the effects and experiences of therapeutic music interventions for individuals with dementia and/or their family care partners (9–12). A recent qualitative systematic review found that shared musical experiences may support dyads’ individual and collective wellbeing through fostering experiences of connection (13). Based on these findings, the authors developed the *Contextual Connection Model of Health Musicking for People Living with Dementia and Their Family Care Partners* (13). The term *musicking* (14) refers to dyads’ engagement in active and passive shared musical activities (15), and *health musicking* (16) is the intentional use of musicking for health. This model outlines factors that impact dyads’ experiences of connection through musicking (the setting, facilitation, changes over time and the supportive aspects of music) and suggests a relationship between these experiences and dyads’ wellbeing. It also presents multiple types of connection experienced through musicking including: connection to self, music, memories, the here and now, each other, shared dyadic identity, original relationship roles, group members, family members, and community. However, there are notable gaps in the literature. Firstly, there is limited research in home-based settings (13, 17) and there is a lack of perspectives from people with dementia (18, 19). Additionally, the *Contextual Connection Model of Health Musicking* has not been tested for validity, warranting further investigation.

In the context of growing demand and the limited number of music therapists, *music therapy skill-sharing* is increasingly recognised as a way to provide timely access to therapeutic music interventions (20, 21). Within skill-sharing approaches, music therapists work with formal and/or informal caregivers to help them use music safely and therapeutically in dementia care (20). Within the literature, the terms *indirect music therapy* and *music therapy skill-sharing* are often used interchangeably (20, 21), however the term *skill-sharing* may be more suitable for family contexts as it allows for the broader use of music to benefit both members of the family dyad. Early research into skill-sharing with family dyads was conducted by Clair (22), Hanser et al. (23) and Baker et al. (24), suggesting mutual benefits for the wellbeing of people with dementia and their family care partners. Building on these findings, the HOMESIDE music intervention was developed within the HOMESIDE randomized control trial (RCT) (25, 26). The HOMESIDE RCT aimed to examine the effects of therapeutic music interventions delivered by family care partners on the behavioural and psychological symptoms of dementia. The HOMESIDE RCT recruited 432 dyads across five countries (Australia, United Kingdom, Germany, Norway, and Poland), and compared music therapy interventions, reading interventions and standard care. The full protocol is presented in a separate paper (25). The current study was a sub-study of HOMESIDE, utilising qualitative data collected through the RCT alongside additional data collected by the authors. To address the noted gaps in the literature, this study aimed to explore the shared musical experiences of dyads participating in HOMESIDE (26) relative to the *Contextual Connection Model of Health Musicking* (13).

## 2. Methods

This study aimed to gain insights into how dyads experienced shared home-based music in the context of HOMESIDE’s 12-week music therapy skill-sharing intervention (26). We examined their experiences during the 12-week HOMESIDE program and in the 3–6 months after HOMESIDE was completed. This paper outlines an abductive and relational-centred approach to a hermeneutic phenomenological analysis of interview and diary data collected from participants. The study gained ethics approval prior to commencement (University of Melbourne; HASS 1: 2022-14112-25430-8).

### 2.1. Music intervention

The music intervention followed the standardised HOMESIDE protocol (26). The HOMESIDE music intervention was designed to support family care partners to use musical activities mindfully to support the person they cared for. Across 12-weeks, dyads received three training sessions with a music therapist online via Zoom (27). In these sessions, dyads were introduced to four musical activities (singing, music listening, movement to music, and instrument-playing) and family care partners were guided to use these activities to support their family members’ needs. These needs included but were not limited to enhancing connection and communication, regulating agitation and mood, and promoting meaningful occupation. Dyads were encouraged to use these musical activities in their home at least two times per week for approximately 30 min per session. Family care partners also received fortnightly follow-up phone calls with the music therapist. These phone calls allowed family care partners to clarify their understanding of the intervention, resolve any issues or negative responses to music, and gain access to further resources. The phone calls were also used to monitor adherence, check on the wellbeing of dyads and provide support as needed. Dyads completed a diary recording their music use across the 12-week program. Author 1 provided the music intervention for the six participants in this study as part of the HOMESIDE RCT.

### 2.2. Participants

Following their completion of the HOMESIDE program, six dyads were invited to participate in this study. Four dyads were spousal couples and two dyads were mother/daughter pairs. Within these, five of the family care partners were female, and one was male. Due to the importance of rapport within dementia research (28–30), we recruited dyads who had worked with author 1 as their music therapist in HOMESIDE. Dyads were identified as potential participants after completing their final HOMESIDE follow-up assessment, at least 13 weeks after their completion of the program. They were invited to participate by a researcher who had no previous relationship with the dyads. All invited dyads agreed to participate and provided written informed consent (or proxy consent where necessary). In keeping with the HOMESIDE inclusion/exclusion criteria, participants also met the following criteria: one member of the dyad had a diagnosis of any type of dementia; the other member of the dyad was a family member or friend of the person with dementia, and not a formal paid caregiver; dyads were cohabiting; dyads scored ≥6 on the Neuropsychiatric Inventory-Questionnaire (NPI-Q) (31); participants did not have a significant hearing impairment that limited their ability to enjoy musical experiences; dyads had access to an internet connection and a computer, smartphone or tablet (25).

### 2.3. Data collection

This study utilised existing qualitative data from the HOMESIDE RCT, including dyads’ diary entries and transcripts from a semi-structured interview conducted in week 12 of the intervention. To gain further insights into their experiences, we invited dyads to participate in a video-recorded *music-based interview* at least 13 weeks after they completed HOMESIDE. This music-based interview was a semi-structured interview that incorporated active and/or receptive music plus dyads’ reflections on their immediate and prior musical experiences. This interview was conducted and recorded by author 1 online via Zoom (27) to align with the HOMESIDE intervention. Further data was collected through reflexive notes completed by author 1. Data was stored securely on a password-protected computer.

### 2.4. Data analysis

Hermeneutic phenomenology was the guiding theoretical framework for the analysis, where we acknowledged our understanding of participants’ experiences as an interpretation influenced by our own perspectives and beliefs (32). Alongside this, a relational-centred research approach (33) was selected to recognise and learn from the relational knowledge developed by author 1 through working with dyads as a music therapist. To maintain rigour within qualitative research, reflexivity was an essential practice (34). Author 1 engaged actively in cycles of reflexivity including introspection, intersubjective reflection and social critique (35) and engaged the other authors in discussions of emerging ideas. An abductive approach was selected to examine the data relative to the *Contextual Connection Model of Health Musicking for People With Dementia and Their Family Care Partners* (13). Within this abductive approach, data were examined in the context of the model, and new insights were also sought from the data (36). We used some techniques drawn from codebook thematic analysis (37) to guide the coding and analysis process from within a hermeneutic (38) and relational-centred (33) position.

The procedure of analysis began with a coding framework based on the *Contextual Connection Model* and author 1 coded the data abductively through a series of 13 hermeneutic cycles (39). This involved a repeated shifting of focus between individual dyads and the larger data set (39). Data was coded both inductively and deductively with detailed notes and memos recorded using MAXQDA 2020 analysis software (40). Following coding, author 1 developed initial themes based on the final codes while considering the *Contextual Connection Model* abductively. These themes were developed and refined through gaining new perspectives of the data and themes using a variety of data engagement techniques including: immersion, zooming in and out, seeking connections, thinking outside the box, and dialogue within supervision with author 2. The final stage of analysis involved writing up the final themes (37).

## 3. Results

This study identified 15 themes that captured how dyads experienced sharing music during and after the HOMESIDE program, and the meanings of these experiences for dyads (see Table 1). Themes are identified as either a common theme (applied to all dyads) or a significant theme (applied to many or most dyads). The themes are interrelated, they interact in multiple and complex ways, and are organised into three global themes: (1) *Experiences were shaped by complex influences*; (2) *A connected musical ecosystem*; and (3) *Music was a resource for wellbeing* (see Table 1). The terms *musicking* (14) and *sharing music* (41) are used interchangeably to capture dyads’ broad use of music and music-related activities (14, 42). The term *affordances* (43) refers to what music offers dyads in their unique contexts. Where examples are provided, the person living with dementia is positioned centrally, with their family care partners identified in relation to them (e.g., Ray’s wife, Lynn).

**Table 1:** Themes and global themes.

Experiences were shaped by complex influences	A connected musical ecosystem	Music was a resource for wellbeing
1. Dyads’ musical experiences were shaped by **who they are**: complex people with complex relationships and histories (common theme)	5. Musicking afforded **moments of connection** for dyads (common theme)	9. Dyads appreciated the **affordances of music** (common theme)
2. Dyads’ musical experiences were influenced by their **ever-shifting context** (common theme)	6. Musicking **revived** and **strengthened** existing connections (common theme)	10. Dyads learned to **intentionally use music** as a resource for wellbeing (common theme)
3. Dyads’ relationship to music **changed over time** (significant theme)	7. Musicking **facilitated new experiences** (common theme)	11. “It’s not a magic pill”: health musicking **did not always “work**”, and it took effort to find what did (significant theme)
4. Dyads benefitted from **personalised, collaborative, and structured facilitation** (common theme)	8. Musicking provided opportunities for dyads to **witness** and **be witnessed** (common theme)	12. Sharing music was **enjoyable in the moment** (Common theme)
		13. Sharing music was **helpful** for dyads (common theme)
		14. Sharing music supported **confidence** for people living with dementia (significant theme)
		15. For some dyads, when music helped, it was **instant, amazing, incredible, fantastic** (significant theme)

### 3.1. Experiences were shaped by complex influences

Dyads’ experiences of sharing music were influenced by who they are (theme 1) and the context they live in (theme 2). This context was continually shifting as dyads experienced changes to their health and life circumstances. Over the course of the HOMESIDE program, dyads’ experiences were further shaped by changes in how they thought about and used music (theme 3). These changes to their relationship with music were fostered through the personalised, collaborative and structured facilitation they received through HOMESIDE (theme 4). These complex and interacting factors influenced how dyads *engaged* with music and how they *interpreted* their experiences.

For instance, dyads responded differently to musicking based on their personalities and relationship dynamics (theme 1). This was highlighted by Ray’s wife, Lynn, who reflected on how they responded differently during a live music concert:

“So while Ray has never been the one to get up and dance, he was jiggin’ in his seat and singing the whole time, I was up dancing.”

Professional identities and cultural backgrounds also influenced dyads’ expectations of musicking. Myrtle associated musicking with relaxed social gatherings due to her Irish background, while Susan’s Chinese background informed her view of singing and dancing as activities reserved for children or professionals.

Dyads’ ever-changing contexts also influenced their experiences (theme 2). Several dyads reflected on the influence of Covid-19, describing musicking as a “replacement” for other activities during Covid-19 restrictions. In the context of dementia as a progressive condition, some dyads needed to adjust their musical activities as their needs changed. For example, as Vicki’s dementia progressed, her husband Geoff increasingly used music to help her feel connected, centred, and calm in the moment.

Across the 12-week music intervention, dyads’ relationship to music changed, further influencing how they experienced musicking (theme 3). Firstly, dyads changed how they *thought about* music as they recognised and appreciated its affordances. RH’s wife, LM, reflected on this in their interview, stating “I’ve enjoyed the whole process as far as understanding about music more and the impacts that music can make on a life.” Dyads also changed how they *used* music. Overall, dyads used music more *intentionally* and more *frequently*, and many dyads integrated musicking into their daily lives. This was shown by Myrtle’s daughter, Kath, who incorporated music into their daily tasks: “Come dinner time, (music) was really helpful for me with preparing food … It certainly helped mum engage in the kitchen.”

All dyads reflected on the helpful role of facilitation in supporting positive musicking experiences (theme 4). In particular, dyads valued the personalised, collaborative and structured nature of the facilitation. The therapeutic relationship and structure of the program were key to maintaining motivation, highlighting the centrality of the scheduled sessions and follow-up phone calls. As reflected by Ray’s wife, Lynn, “I think it would be very easy, if you were not contacting us, it would be very easy just to fall off the wagon.” The personalised and collaborative aspects of facilitation enabled dyads to find solutions to challenges they encountered at home, and helped them identify which activities were most helpful and/or suitable. Richard’s wife, Meg, reflected on this in their interview:

“The (musical activities) that you came up with were so pertinent to us. I think you listened, and you knew something about us and knew what we were capable of. So you came up with the idea (of songwriting) … Your coaching us was really, really beneficial.”

### 3.2. A connected musical ecosystem

Dyads experienced multiple levels of connection through musicking as they connected to themselves, each other, friends, family members, and sometimes the broader community. Dyads’ experiences of connection were experienced temporally as *moments* (theme 5). Within these moments, dyads experienced the revival and strengthening of their existing connections to self, memories and each other (theme 6). Some dyads also developed new connections through songwriting, learning new songs, and making new social connections beyond the home (theme 7). Additionally, sharing music provided opportunities for dyads to experience each other relationally, inhabiting familiar and new roles, and understanding themselves within an ecological context as they witnessed others and were witnessed themselves (theme 8).

Vicki’s experiences of sharing music with her husband, Geoff, highlight the temporal and meaningful nature of moments (theme 5). While Vicki experienced difficulties with focus and verbal communication, she often became more engaged and animated during musicking. For Vicki, this was meaningful and enjoyable in the moment, as she appeared to connect with Geoff through smiling, increased eye contact, and physical affection. For Geoff, while these moments were “very much few and far between,” he valued the “rekindling” of their connection, reflecting that “anything is a blessing.” This rekindling or reviving aspect of connection was a common experience across dyads (theme 6). Through sharing music, dyads revived and strengthened existing connections to music, memories, each other, the self, memories and the *ability* to remember. These reconnections were meaningful to dyads in the context of lost connections due to symptoms of dementia.

Musicking supported people with dementia to reconnect to a sense of self, and re-identify with their musical identities. For instance, Susan reflected that she enjoyed singing, reflecting on her role as “lead singer” in her middle and high school classrooms. Others, such as RH, viewed music as “just part of my life.” All dyads experienced reconnections to memories through musicking, and reminiscing became a rich source of conversation for many. This supported reconnections to each other as dyads, particularly for spousal couples, who revisited positive shared memories through music.

Musicking also supported *new* connections for dyads. This included connecting to music in new ways through various HOMESIDE activities, and creating new neural connections through songwriting, learning songs, building social connections and creating memories. The creation of new memories was particularly meaningful for family care partners as they recognised the ongoing value of these. Myrtle’s daughter, Kath, reflected on the significance of new memories, as Myrtle passed away before their final interview. Kath reflected:

“There is one image that always (comes back), Mum and I just sitting on the couch and laughing and she just put her hand out, and we’d hold hands just quite naturally. And that’s something that she did a lot when the music was playing … My mum just hold(ing) my hand is such a simple gesture, but … it’s invaluable to me. It’s a beautiful memory to have.”

Sharing music also allowed dyads to experience each other relationally as they witnessed each other playing, singing and remembering (theme 8). Family care partners experienced pleasure at seeing their loved ones’ strengths through music, frequently commenting on their ability to remember songs and lyrics. Being witnessed was an equally powerful experience as participants with dementia were seen through their strengths (theme 8). The dynamic of witnessing and being witnessed through music also highlighted dyads’ relational knowing of each other, with each able to comment on the preferences of the other. This provided opportunities to recapture original relationship roles as demonstrated by Richard and his wife Meg:

**Meg:** The dancing. I love dancing. I loooooooove dancing.

**Richard:** Oh you sure do.

It is important to note that witnessing and being witnessed was not always a positive experience. Musicking also reflected changes to people with dementia’s abilities as their condition progressed, and this was sometimes challenging for dyads. For example, as Vicki became less responsive to music over time, her husband Geoff reflected that this lack of response was “fairly representative of where she’s at the moment.” This made witnessing challenging for Geoff as he expressed grief around changes to Vicki’s abilities.

### 3.3. Music was a resource for wellbeing

As dyads changed how they thought about music (theme 3) they gained an appreciation of its affordances (theme 9), drawing on these more intentionally over time (theme 10). While it sometimes took effort to find what “worked” (theme 11), musicking was broadly experienced as enjoyable (theme 12) and helpful (theme 13). As musicking empowered people with dementia to express and build on their strengths, they became more confident in their abilities (theme 14). Family care partners also gained confidence in their caregiving role (theme 14). While musicking was not *always* helpful, at times it was experienced as “instant,” “amazing,” “incredible” and “fantastic” (theme 15).

While the affordances of musicking varied due to dyads’ individual contexts, music was broadly identified as flexible, accessible and adaptable. This afforded opportunities for social connection, positive mood, reduced distress and improved cognitive abilities (e.g., the ability to remember; theme 9). Several people with dementia commented on music’s capacity to support their memory, linking this to improved self-esteem and confidence. Ray commented on this in his interview:

“It’s good having an ability where I can (remember songs) … (I’ve) still got to go back into the (memory) files, they are not always there, I do not pull the right one up all the time, but the list is still there.”

The soothing capacity of music was also valued, with several family care partners reflecting on using music to soothe their loved ones and themselves. Dyads’ intentional use of music increased as their participation in the HOMESIDE trial progressed (theme 10), and this was reflected in their interviews and diaries. Dyads used music intentionally to interact and bond with each other, to soothe, to “use the brain” (Richard), and to manage mood and stress. As dyads became more comfortable with using music as a resource for wellbeing, they were able to use it more flexibly as needed, with some integrating musicking into their daily lives.

Importantly, using music for wellbeing did not always ‘work’ for dyads, and it sometimes required effort to discover what was helpful (theme 11). Definitions of what ‘working’ meant varied across dyads, and this was often influenced by their expectations of music for dementia. For example, two family care partners referred to videos they had seen on social media of people with dementia’s dramatic responses to music. At times, family care partners used these videos as a comparison point for their loved ones’ less dramatic responses, which resulted in some disappointment. Susan’s daughter Jenny reflected on this more moderate impact of musicking for her mother: “So music has eased its way in to give a little bit of pleasure. It’s not a magic pill.” For other dyads, it took a process of trial and error to find which musical activities were most helpful. Dyads valued supportive facilitation within this process to help with problem-solving and motivation.

Despite these challenges, musicking was broadly experienced as enjoyable in the moment (theme 12). This was discussed within diary entries and interviews and observed during music-based interviews. In the longer term, sharing music was experienced as helpful for dyads’ mood (theme 13). Most people with dementia also experienced music as helpful for their memory, and some family care partners felt better able to cope with the challenges of caregiving (theme 13). Myrtle’s daughter, Kath, reflected on several helpful aspects in her interview:

“Being able to diffuse a stressful situation early on in the piece with music had undoubted benefits for me. But also the happiness part of it, that’s good for you as well. When we got into the study, we both had a bounce in our steps.”

Knowing how to use music as a caregiving tool boosted the confidence of some family care partners. At the same time, musicking supported people with dementia’s confidence as it reinforced their strengths such as memory and musical abilities. This was shown by Ray, who reflected that remembering is “a bit of an achievement sometimes.” This increased confidence supported musical flourishing for two participants with dementia as they embraced musicking as a creative expression of self. With newfound confidence, their self-expression expanded into performing for other family members and in community spaces.

While musicking did not always ‘work’, at times it was notably impactful and affecting (theme 15). These moments were highly valued by dyads, providing a welcome contrast to experiences of loss and decline. These peak moments captured what is possible for dyads through musicking while serving as a reminder of people with dementia’s continuing strengths. For people living with dementia, these are moments of pride, joy and hope. For family care partners, these experiences are furthered through the creation of cherished memories. One of these moments was captured through author 1’s observational notes from Richard and his wife Meg’s music-based interviews:

“In this moment, Richard appears to be completely in the moment with the music. There is no hesitation in his movement, just playfulness and confidence. He is confident in his abilities, and in expressing himself. Meanwhile, Meg also seems to be connecting to the music as she joins in and sings, laughing intermittently. These laughs appear to be joyous, and as she later emails me, she gets joy from watching Richard in his confident self-expression.”

## 4. Discussion

**Figure 1:**
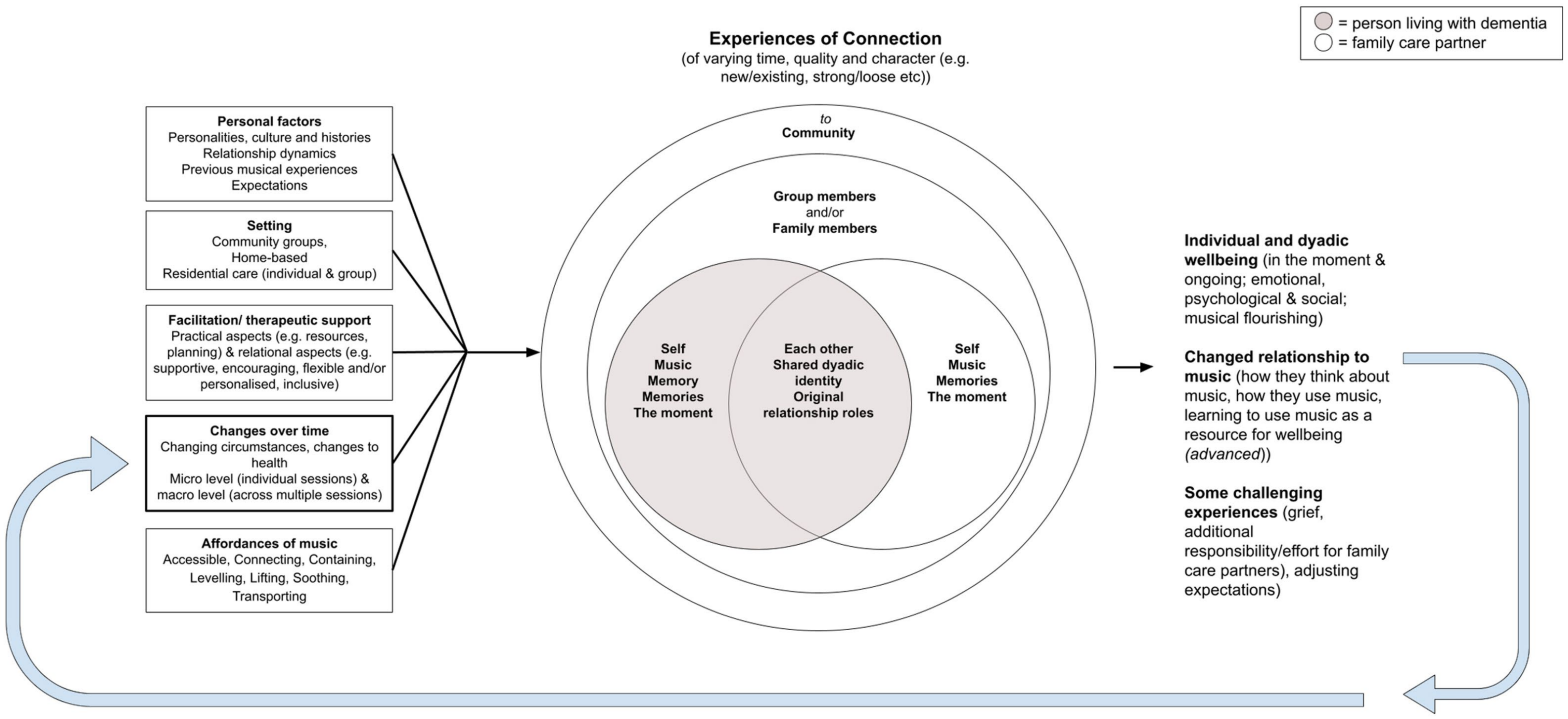
Revised contextual connection model of musicking for people living with dementia and their family care partners.

The findings of this study shed new light on the *Contextual Connection Model of Health Musicking for People With Dementia and Their Family Care Partners* (13), indicating the need for several changes. Most notably, dyads’ experiences of connection through musicking were newly understood as multifaced, ecological and cyclical. This understanding aligns with Ansdell’s (15) concept of *musical worlds*, which views musical encounters as ecological and contextual. Additionally, this study highlighted the influence of personalities, relationships and histories on dyads’ experiences of connection through music. We also identified challenging experiences such as grief, adjusting expectations and the effort required to use music effectively at home. Further, author 1’s understanding of the *supportive aspects of music* evolved to align with concepts of *musical affordances*, where music is understood to offer different opportunities depending on the context (43, 44). To reflect these new understandings, a *Revised Contextual Connection Model of Musicking for People Living with Dementia and Their Family Care Partners* was developed (see Figure 1). The change in terminology from *health musicking* (16) to *musicking* (14) reflects renewed understandings of dyads’ intentionality during musicking, where it is recognised that dyads do not *always* use music deliberately to support their health. This revised model reflects the cyclical nature of dyads’ experiences of connection through music, as each new musical encounter is shaped by their ever-changing context. We newly frame dyads’ experiences of connection within an ecological framework (45), highlighting the interconnected and multifaceted nature of connections. Further, the effects of these connections are expanded, showing that challenging experiences and changes to dyads’ relationship to music may occur simultaneously with and/or alongside improved wellbeing. Within this model, *learning to use music as a resource* is identified as an advanced outcome facilitated through personalised and focused interventions (such as HOMESIDE). While it is possible that dyads may develop greater understandings of musical affordances through participation in other settings (such as community music groups or traditional music therapy), we argue that the development of the conscious use of music requires more focused support.

### 4.1. Conceptualising connection

The results of this study present new insights into dyads’ experiences of connection through musicking across settings, plus nuanced understandings of dyads’ experiences of connection within home-based settings. Based on these findings, we conceptualised connection through musicking for dyads as: (1) influenced by context; (2) ecological; (3) temporal; (4) embodied; (5) of varying quality and characteristics; (6) supported by the affordances of music; (7) supportive for dyads’ individual and collective wellbeing; and (8) not a magic pill.

This paper has a specific focus on discussing the music therapy practice implications of a home-based skill-sharing music intervention for dyads. Therefore, this discussion primarily focuses on the findings relating to the advanced outcome of *learning to use music as a resource for wellbeing* presented in the *Revised Contextual Connection Model of Musicking for People Living with Dementia and Their Family Care Partners* (Figure 1).

### 4.2. Learning to use music as a resource

The findings of this research highlighted the numerous affordances of musicking for dyads. Musicking may afford opportunities for soothing, connecting, lifting mood and abilities, and offers multiple entry points for increased accessibility. However as argued by Ansdell (15), making use of these affordances is not an innate skill. It must be learned or facilitated by others. This research showed that dyads participating in HOMESIDE learned to use music as a resource with the support of personalised, collaborative and structured facilitation. This supports findings of previous studies where dyads learned to use music to support their practical and emotional needs in their daily lives (17, 46, 47). These studies also suggest positive outcomes for dyads learning to use music as a resource, such as improved coping for family care partners, and improved emotional wellbeing for both members of a dyad. However, insights into the *learning* process for dyads have not previously been explored. The current study contributes novel findings to this area by illuminating core aspects of dyads’ process of learning to use music as a resource. This included: (1) changes to how they think about music, (2) appreciating the affordances of music, (3) learning to use music intentionally, (4) trial and error, (5) adjusting expectations, and (6) the importance of personalised, collaborative and structured facilitation.

Through participating in HOMESIDE, dyads changed how they thought about music as they gained an appreciation of its affordances through a process of trial and error. Changes in how dyads thought about and used music occurred gradually as they learned through doing and reflecting on their experiences through supported facilitation. The active nature of appropriating the affordances of music is discussed by DeNora (48), who describes the use of music in daily life as recruiting a *technology of the self*. For healthy individuals, appropriating music is largely sub-conscious, but in the context of illness, the use of music to support health may require a more conscious approach (48). This intentional use of music for health is defined as *health musicking* (16). There is limited research examining how dyads may develop an intentional approach to musicking, however Batt-Rawden and Tellnes (49) provide some insights into the skill-development process for people with long-term illness. In their longitudinal ethnographic study, Batt-Rawden and Tellnes investigated how 22 participants engaged with a selection of musical CDs with no formal intervention provided. They describe how participants learned to appropriate music through increasing their awareness of themselves and of music’s affordances. This aligns with our findings on the importance of increased awareness in the learning process. In contrast to HOMESIDE, Batt-Rawden and Tellnes’ study discusses “indirect learning,” leading to findings that knowledge was acquired “obliquely … (and) in ways that elude conscious processing” (49). The authors also argued that this learning required extended periods of time. The current research points to the value of a more direct approach to learning, as we found that all dyads learned to use music as a resource over a 12-week period with the support of a direct skill-sharing approach.

A personalised and collaborative approach was also key to supporting dyads through a process of trial and error as they learned which musical activities were most helpful for their needs. To our knowledge, this process of trial and error has not been identified in previous skill-sharing research, particularly with family dyads. Within this process, dyads valued the expertise of the music therapist who was able to provide personalised and timely musical ideas and information about the common affordances of musicking. This aligns with broader research that identifies timely and personalised information as a core need for family care partners (50). Dyads also valued the therapeutic relationship with the music therapist, sharing this helped them maintain motivation and manage the challenging aspects of the learning process. These challenges included experiences of grief, disappointment and adjusting expectations. In a parallel study investigating HOMESIDE participants’ experiences in Norway, Stedje et al. (47) also identified the vulnerabilities dyads face as they learn to use music in their homes, suggesting music therapists may be essential supports in this context. Music therapists may be particularly well-equipped to provide skill-sharing for family dyads who have dual needs of education and emotional support. As noted by McDermott et al. (20) supportive interventions for family dyads may oscillate between direct and indirect music therapy approaches as the needs of the dyads change. Music therapists are uniquely trained in responding to the individual and changing needs of clients (51) and specialise in the use of music for achieving non-musical outcomes. Therefore, to manage the complex needs of family dyads, music therapists may be the most appropriate facilitators for home-based interventions. To support dyads in learning to use music as a resource, therapists should consider the following aspects: the importance of personalised facilitation, the process of trial and error, maintaining motivation, dyads’ expectations, the context of continual change, and opportunities to connect dyads to the community. These are discussed below.

### 4.3. Supporting dyads to appropriate music

#### 4.3.1. Personalised facilitation

As found in this research, dyads benefit from personalised facilitation to meet their complex and changing needs. This aligns with the broadly accepted recommendations for the personalised use of music for people with dementia (52, 53) and the need to consider family dynamics within family-centred approaches to dementia care (54, 55). Due to the heterogeneity of dyads’ contexts, music therapists need to build an understanding of dyads’ *musical worlds* (15) to provide the best individualised care. As the results illustrate, these musical worlds are influenced by dyads’ personalities, relationship dynamics, previous musical experiences, culture, the setting, and dyads’ changing health and circumstances. In practice, much of this information may be gathered during the initial assessment, however an understanding of dyads’ relationship dynamics may build over several sessions. Changes to health and circumstances must be continuously assessed. Additionally, in alignment with findings by Dassa et al. (17) and Stedje et al. (47), this research highlights the complex and different needs of family care partners within the dyadic context, indicating the need for awareness and sensitivity around dementia grief (56). While the importance of supporting dementia grief within dyadic contexts is identified in several studies, there is limited discussion on how music therapists might best do this. Melhuish et al. (46) identify the relevance of Blandin and Pepin’s framework (56) for music therapists supporting dementia grief in dyadic contexts, however they do not elaborate on how music therapists might incorporate this into practice. Within this research, the creation of new memories played a role in supporting grief for family care partners while some experiences may have highlighted their loved ones’ decline. This suggests further research is needed to understand how family care partners experience dementia grief within dyadic interventions, as well as how music therapists might best support them to process and adapt (56).

#### 4.3.2. A process of trial and error

Personalised facilitation is particularly valuable for supporting dyads through a process of trial and error to identify which musical activities are most helpful. As dyads are introduced to new musical activities, their process is facilitated through experiential learning (57) with opportunities for reflection, validation, support to refine activities, motivation and emotional support. The use of experiential learning with opportunities for reflection was effectively used by Beer (58) to train formal caregivers in the therapeutic use of music. The experiential nature of learning is also identified by Ridder and Bøtker (59) in providing Person Attuned Music Intervention (PAMI) training for professional caregivers, who note that “knowledge may seem irrelevant until they experience *how* it works” (59). Krøier et al. (60) also identify helpful areas of focus for learning to attune through music: tempo, timing, and dialogue. For family dyads, this learning process is made more complex by the personal nature of the proposed intervention, and the challenges of integrating intentional approaches to music in the home-based setting. Music therapists are well-equipped to provide validation and emotional support through the therapeutic relationship, and have well-developed skills in adapting musical activities (59). Therefore, music therapists are particularly well-suited to provide music therapy skill-sharing within a family dyadic context as they can support experiential learning with additional problem-solving and emotional support elements.

The pace of introducing activities is another important consideration. In facilitating HOMESIDE, it was helpful to focus on one or two activities per session to avoid information overload. The flexible design of HOMESIDE enabled tailoring the pacing to meet the needs and preferences of dyads (26), and this also aligns with the PAMI training manual for professional caregivers (61). This finding highlights the importance of flexible skill-sharing approaches that allow the pacing to be personalised for each dyad.

#### 4.3.3. Supporting motivation

Study findings also highlighted the importance of structure and external accountability as a source of motivation for dyads. Dyads appreciated the regularity of sessions and phone calls and the reflective nature of completing a diary. The enjoyable nature of musicking was also experienced as motivating, which aligns with previous findings in the literature (18, 62, 63). Within the home-based setting, some family care partners found it difficult to maintain motivation to use music after the program ended, despite their desire to utilise its affordances. This has implications for the sustainability of skill-sharing programs, suggesting dyads may benefit from ongoing support to maintain motivation in the longer term. Considerations of sustainability are imperative for good music therapy practice (64), and have been noted as an area for development in dyadic music interventions (46, 65, 66). While skill-development and knowledge transfer provide a strong foundation for dyads’ ongoing use of music, this foundation may need bolstering through additional supports. This aligns with findings by Dassa et al. (17) who specifically explored the sustainability of a home-based musicking program. While Dassa et al. (17) found that the sustainability of an intensive 12-week program was promising, they recognised that one of their participating dyads (out of a sample of two dyads) needed ongoing support due to the progression of dementia. To support this need, they provided continuing monthly sessions and fortnightly phone calls for the dyad. The ongoing sustainability of their approach was not reported on (17).

Bolger and McFerran (64) suggest multiple elements to support the sustainability of music therapy programs, including links to independent ongoing programs, skills/knowledge transfer, links to wider community, and the provision of resources. For dyads learning to use and maintain musicking as a resource for wellbeing, connections to community music groups and the provision of resources may be indicated alongside the provision of ongoing individual supports. Further research into these ongoing supports is needed to assess their feasibility and effectiveness.

#### 4.3.4. Understanding expectations

Alongside this process of trial and error, therapists should aim to understand dyads’ expectations around the challenges and benefits of musicking. As highlighted through this home-based study, dyads’ expectations may be influenced by videos shared through social media, and these may not realistically reflect what musicking affords within daily life in the context of dementia, nor the effort required to appropriate these affordances. Unrealistic expectations may lead to disappointment for dyads; therefore, music therapists should endeavour to present realistic expectations in public and private communications. Additionally, it may be important to validate and support dyads to adjust these expectations as they may be linked to dementia grief (67). Little has been written about the influence of media on expectations around music and dementia, however critics of media in the broader disability space suggest that viral videos are typically shared without context, and may create unrealistic expectations of people’s abilities (68). At a broader level, discussions around client expectations of music therapy are scarce across populations, indicating the need for more research in this area.

#### 4.3.5. Adjusting to continual change

Dyads experience musicking in the context of continual change. For music therapists, this requires ongoing adaptability and knowledge of symptoms and interventions across all stages of dementia. While music therapists must be prepared for future changes, providing information that is not focused on dyads’ current needs and situation may cause unnecessary stress (69). Therefore, it is recommended that interventions focus on supporting dyads in the moment while simultaneously building resources that may be helpful for dyads in the future. These resources might include the development of playlists, musicking routines, and connections to community. This approach aligns with resource-oriented aspects of Community Music Therapy (70) which focus on developing strengths, material resources and social/relational capital. Due to the progressive nature of dementia, dyads may also benefit from access to ongoing support or ‘top-up’ sessions to help them adjust their use of music over time. As previously discussed, further research is needed to identify which types of support might be most beneficial at different stages of the dementia journey for dyads. This research may extend to exploring the role of musicking in bereavement after a family member has died.

#### 5.3.6 Connecting to community

Supporting dyads to connect to community through music may provide multiple benefits. As found through our earlier thematic synthesis, group musicking provides strong social supports and fosters meaning-making, personal growth, flourishing and citizenship. These aspects contribute to expanded notions of wellbeing for people living with dementia that go beyond coping and surviving (71). Additionally, as highlighted within our home-based research, dyads may benefit from support to maintain their use of music following a period of focused training. Connecting dyads to peers through community musicking groups or online support discussions may function to support motivation and adaptation over the longer term. This supports findings by Clark et al. (63) where the enjoyable aspects of group therapeutic songwriting motivated dyads to “do more with music” beyond sessions.

While some dyads may struggle to attend group musicking in person due to difficulties leaving the house or geographical remoteness, online options are increasingly available due to responses to Covid-19 (72). While there are challenges and limitations in conducting group musicking in online settings (e.g., latency, synchronicity and hesitance/anxiety in using technology) (73), a growing number of studies show that online group musicking is both feasible and beneficial for dyads (74–76). Research shows dyads are increasingly confident and comfortable using technology (73), and online group musicking may offer an accessible option for reducing social isolation and supporting mood (72, 75). As a recent addition to practice, further research is needed to examine the barriers and benefits of online group musicking for dyads.

To further support connections to the community, music therapists may also consider broader music networks available to dyads, such as local concerts, churches, singing groups, workshops and other community music offerings. As found by Hara (77), community music groups may serve as a central activating point for musical and extra-musical care pathways, supporting citizenship for dyads through integrated connections to community.

### 4.4. Strengths and limitations

This study provided unique insights into music therapy skill-sharing, a developing area of practice, presenting practical and theoretical considerations for music therapists and program developers. The innovative use of video-recorded music-based interviews provided opportunities for people with dementia to share their experiences through musical and non-verbal communication, and these data were strengthened through triangulation with diary and interview data across multiple time-points. The use of video-recording via Zoom (27) also limited our observations to what was captured on the screen. Therefore non-verbal responses outside this frame (such as toe-tapping, fidgeting etc.) were potentially missed. This may have influenced our interpretation of dyads’ experiences. While this study offers some insights into dyads with adult children as care partners, more research is needed to identify differences in needs between spousal and adult/child dyads. This study also had limited diversity as all dyads lived in Australia, most participants were born in Australia and spoke English as their first language, and all spousal couples were heterosexual. Future studies would benefit from greater diversity to understand dyads’ experiences in different cultural contexts.

## 5. Conclusion

This study offers new insights into dyads’ experiences of sharing music across settings, informing the *Revised Contextual Connection Model of Musicking for People Living with Dementia and Their Family Care Partners*. This study also presents insights into dyads’ process of trial and error as they learned to use music as a resource. To support this process, dyads benefitted from personalised support to overcome challenges, maintain motivation, adjust to change and connect to community. Music therapists play a crucial role in providing both practical and emotional support for dyads to identify and utilise the affordances of music. Dyads may benefit from follow-up support to adapt as dementia progresses, and connections to broader resources in the community. Importantly, while musicking is largely helpful and enjoyable for dyads, music therapists need to be sensitive to expectations around the benefits of music. While this research provided new insights into dyads’ experiences of using music as a resource, further research is needed to understand what approaches are most helpful at different stages, and the role of musicking in dementia grief and bereavement. Additionally, research into ongoing support for dyads is needed, including ‘top-up’ supports, connections to peers, and musical and community-based resources.

## Data availability statement

The raw data supporting the conclusions of this article will be made available by the authors, without undue reservation.

## Ethics statement

The studies involving human participants were reviewed and approved by University of Melbourne, HASS 1 ethics committee. The participants provided their written informed consent to participate in this study. Written informed consent was obtained from the individual(s) for the publication of any potentially identifiable images or data included in this article.

## Author contributions

KMcM led the design, implementation, analysis, writing and editing of this study, and drawing on the HOMESIDE music intervention protocol (26). FB and KMcF provided supervision for KMcM across these processes. All authors contributed to the article and approved the submitted version.

## Funding

This work was supported by the University of Melbourne, National Health and Medical Research Council (APP1169867) and the EU Joint Programme—Neurodegenerative Disease Research (JPND/04/2019).

## Conflict of interest

The authors declare that the research was conducted in the absence of any commercial or financial relationships that could be construed as a potential conflict of interest.

## Publisher’s note

All claims expressed in this article are solely those of the authors and do not necessarily represent those of their affiliated organizations, or those of the publisher, the editors and the reviewers. Any product that may be evaluated in this article, or claim that may be made by its manufacturer, is not guaranteed or endorsed by the publisher.
